# Effects of Ion Irradiation on Seedlings Growth Monitored by Ultraweak Delayed Luminescence

**DOI:** 10.1371/journal.pone.0167998

**Published:** 2016-12-09

**Authors:** Rosaria Grasso, Tomoko Abe, Giuseppe A. P. Cirrone, Giacomo Cuttone, Marisa Gulino, Francesco Musumeci, Francesco Romano, Hiromichi Ryuto, Agata Scordino

**Affiliations:** 1 Department of Physics and Astronomy, Catania University, Catania, Italy; 2 Laboratori Nazionali del Sud, Istituto Nazionale di Fisica Nucleare - Catania, Italy; 3 RIKEN Nishina Center, Hirosawa, Wako, Saitama, Japan; 4 Facoltà di Ingegneria e Architettura, Università di Enna Kore, Enna, Italy; 5 Photonics and Electronics Science and Engineering Center, Kyoto University, Kyoto, Japan; United States Department of Agriculture, UNITED STATES

## Abstract

The optical technique based on the measurement of delayed luminescence emitted from the biological samples has demonstrated its ability to provide valid and predictive information on the functional status of various biological systems. We want to extend this technique to study the effect of ionizing radiation on biological systems. In particular we are interested in the action of ion beams, used for therapeutic purposes or to increase the biological diversity. In general, the assessment of the damage that radiation produces both in the target objects and in the surrounding tissues, requires considerable time because is based on biochemical analysis or on the examination of the evolution of the irradiated systems. The delayed luminescence technique could help to simplify this investigation. We have so started our studies performing irradiations of some relatively simple vegetable models. In this paper we report results obtained from mung bean (*Vigna radiata*) seeds submitted to a ^12^C ion beam at the energy of 62 MeV/nucleon. The dry seeds were irradiated at doses from 50 to 7000 Gy. The photoinduced delayed luminescence of each seed before and after ion irradiation was measured. The growth of seedlings after irradiation was compared with that of untreated seeds. A growth reduction on increasing the dose was registered. The results show strong correlations between the ion irradiation dose, seeds growth and delayed luminescence intensity. In particular, the delayed luminescence intensity is correlated by a logistic function to the seedlings elongation and, after performing a suitable measurement campaign based on blind tests, it could become a tool able to predict the growth of seeds after ion irradiation. Moreover these results demonstrate that measurements of delayed luminescence could be used as a fast and non-invasive technique to check the effects of ion beams on relatively simple biological systems.

## Introduction

In recent years the treatment of biological systems via ion beams has opened new opportunities in some important applicative fields, in particular in cancer treatments [1–3] and in enhancement of biological diversity, obtained by inducing mutations in seeds [4–7]. Even if many studies are present in literature, the basic mechanisms underlying biological effects and the real effectiveness of ion beam in the target objects and in the surrounding volume are not well clarified [8–13]. At present, the techniques used to obtain information on the status of the treated systems are characterized by high invasiveness, long times and high costs. So an early non-invasive diagnostic technique, capable to give information on the status of the treated systems, would be welcome.

The increasing sensitivity of recent optical techniques allows the development of non-invasive tools to explore the state of biological systems. Indeed, photons are able to probe the chemistry and the physical structure of biological systems, providing information useful to disentangle their complexity. For these reasons, optical techniques, as fluorescence or reflectance spectroscopy, have been applied in various fields [14–17].

More recently, some reports have demonstrated that the ultraweak photon emission from the biological systems is able to provide information on the state of the organisms and on several organic processes [18]. In particular, by using the delayed luminescence (DL), also called delayed fluorescence, in the study of human cells, the possibility to discriminate between normal and tumor conditions [19], or to reveal the activation of the apoptotic pathway, induced by chemical agents [20–22], has been established. Moreover as it regards the seeds, it has been shown that the vegetative performances of artificially aged seeds are related to the characteristics of DL measured on dry seeds [23–24].

The DL is the ultra-weak emission of optical photons, subsequent to excitation via light. It lasts in time up to seconds. This phenomenon is quite widespread in biological systems, and it has also been observed in condensed matter systems. In both cases, it seems to derive from the microscopic structure of the sample [25]. This dependence has been justified, within the condensed matter theory, by charge recombination processes, which proceed through a metastable state, taking into account the possibility of generating collective excitations in the molecular structures that constitute the organic matter [26–27].

In this paper the possibility to use DL measurements to monitor the effects of heavy ion beams has been investigated by choosing, as biological sample, dormant seeds, due to their stable (not developing) stage, both before and after the ion irradiation. This stability of the system allows obtaining reproducible DL measurements while time elapses.

On the other hand the importance of seeds in the evolution of life on our planet is widely recognized and recently the possibility to induce several kinds of genetic mutations in seeds by using high-energy ion beams has been demonstrated [4–7].

As above said, it is possible to check the effect of treatments only after seeds germination. Due to biological diversity and to the relative low probability of mutation, to get information about the evolution of dormant seeds, i.e. to identify the mutated samples inside the irradiated set, is appealing.

So we have measured the DL of dry mung bean seeds, after irradiation with a 62 MeV/nucleon ^12^C^5+^ beam at different doses. DL features were correlated with both the ion dose values and the growth parameters of the seeds, once germinated.

The results reveal interesting correlations, so confirming the prospective capability of DL measurements in the field of quality discrimination technique.

## Materials and Methods

### The biological material

Samples used in this work were mung beans (*Vigna radiata*) seeds, coming from organic agriculture and stored at room temperature. Seeds had a prolate spheroid shape and were selected in order to have similar sizes: about 5 mm × 3 mm. Their average weight was 74 ± 6 mg. The germination capability of native seeds (220 seeds were analyzed) was higher than 99%. When a seed germinated, the primary root or radicle appeared two days after the sowing, and in six days all seedlings had fully developed unifoliate leaves and had an average length of 196±8 mm).

Overall 484 mung beans seeds were used, 264 of which were exposed to ion irradiation at different doses, as reported in Table 1, during two different irradiation runs.

**Table 1:** Number of seeds for germination and DL test at every ion beam dose.

Ion beam Dose (Gy)	No of seeds for germination test	No of seeds for DL test
0*	220	132
50	36	24
100	24	24
300	24	24
500	36	24
1000	60	24
5000	48	24
7000	36	36

*0 Gy dose value refers to untreated seeds (i.e. control)

After irradiation, DL emission from single seed, by using a representative number of seeds, was measured, completing the entire set of DL measurements in two weeks. After that, the growing tests started, grouping irradiated and untreated seeds (see. Par. 2.3) in batches. The growth tests were completed in two months. Because seeds remained dry and under controlled conditions for all the time before the planting process, we assumed that no significant metabolic events occurred in this time, so considering different batches as if they were in the same conditions.

### Ion beam irradiation

**Fig 1:**
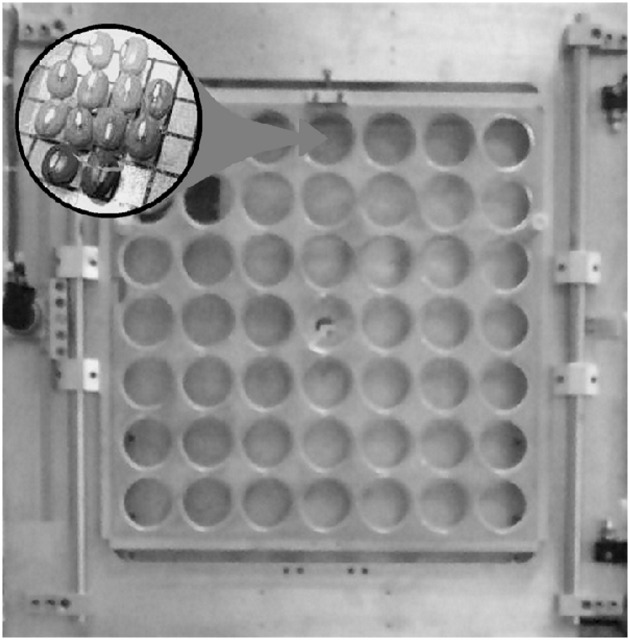
Remote controlled irradiation system. Photograph of the remote controlled irradiation system. During ion beam exposure each hole contained a sample holder with 12 seeds according to the arrangement shown in the inset.

The seeds were irradiated on air by using a ^12^C^5+^ ion beam delivered by the K800 Superconducting Cyclotron of the Laboratori Nazionali del Sud, INFN (LNS-INFN, Catania, Italy) at energy of 62 MeV/nucleon, that is intermediate with respect to the high (about 100 MeV/nucleon) and the low (few keV/nucleon) energy ranges mainly used. The ions were extracted from the vacuum beam pipe through a window made of a 50 μm thick kapton foil, passed through a ionization chamber, used for dose evaluation, and reached the irradiation position, placed at 115 cm from the beam pipe exit window. The beam spot at the irradiation position was 20 mm in diameter, with 85% uniformity. According to the calculations performed by LISE++ code, taking into account the energy lost by the ions on going through materials, at the irradiation position the physical properties of the beam were the following: total residual beam energy: 678 MeV, LET equal to 61 ± 1 keV/μm, penetrating depth in water 9.2 mm. The sufficiently high beam energy allowed achieving an almost uniform dose distribution on seed depth, avoiding the effect of the Bragg peak and the ions implantation inside the seed. The ionization chamber was calibrated by comparison with the plane-parallel advanced PTW 34 045 Markus Ionization Chamber, usually adopted for reference dosimetry. The beam calibration was performed just before each irradiation run (about 10 hours). As the dose variation (in monitor unit) in four consecutive days resulted within 3%, the error on adsorbed dose measurement was lower than 1%. An automatic system was developed in order to stop the beam by using a mechanical valve when the monitor units in the proportional chamber matched the settled value. The samples were put on the irradiation position by means of a remote controlled irradiation system, which allowed to automatically irradiate in air a large number of samples and reduce dead time for samples’ positioning. A photo of the set-up is shown in Fig 1. It consisted essentially of two parts: a frame that could be moved in horizontal and vertical directions (x and y) and a sample charger, which was easily fixed at the frame. The seeds were set in suitable holders, each containing 12 seeds arranged in such a way that all the seeds were evenly irradiated. Moreover, also the position of each seed with respect to the beam spot was fixed (see inset of Fig 1). By moving the irradiation system, each batch containing 12 seeds was placed in the ion beam position, until the settled dose was delivered on it. A shield with a collimator of 20 mm in diameter defined the irradiation zone, avoiding the irradiation of the seeds contained in the neighbor holders.

Doses of 50, 100, 300, 500, 1000, 5000 or 7000 Gy were deposited on seeds. Table 1 reports, for each ion dose, the number of irradiated seeds whose growth was measured. The line corresponding to 0 Gy dose value refers to untreated seeds (i.e. control).

### Growing measurement

**Fig 2:**
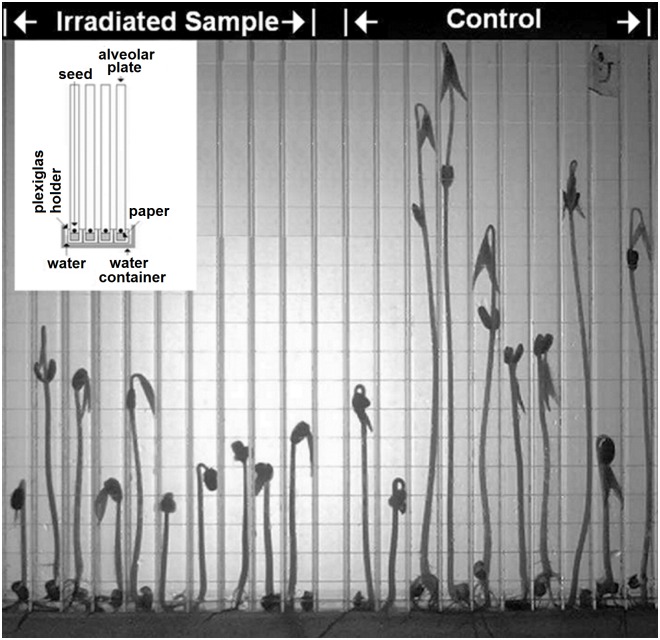
Alveolar plate for seeds' growth. Photograph of the polycarbonate alveolar plate where the seeds were grown under controlled conditions. In the inset, the scheme of the plates’ arrangement inside the thermostatic chamber is shown.

The growth of the seeds was carried out under controlled conditions inside a chamber, provided with a forced ventilation unit and characterized by a temperature of 25 ± 0.5°C and a relative humidity of 60 ± 5%. Each seed was placed inside a single cell (dimensions: 1cm × 1cm × 23cm) of a polycarbonate alveolar plate (see Fig 2), containing the whole group of 12 seeds of each irradiated batch, and 11 untreated seeds as reference.

The cells bottoms were closed with a rolled up sheet of filter paper and inserted in a Plexiglas framework. The plates were partially dipped into distilled water contained in a suitable vessel (see inset in Fig 2).

Temperature and humidity values were recorded with two Escort Logger (mod.60D32), each with 0.3°C accuracy and minimum resolution 0.1°C, placed as close as possible on the left and right side of the water containing vessel.

Growth was monitored regularly by catching images of the plates every 24 hours. An example is shown in Fig 2.

The length of every seedling was calculated from the pictures and the entire procedure carried up for six days.

### Delayed luminescence measurements

The delayed luminescence of seeds was measured by using an improved version of ARETUSA set-up developed at LNS-INFN and able to study the spectral properties of the DL issued by individual seeds [21].

In brief, the excitation source was a pulsed Nitrogen laser (λ = 337 nm, pulse width 5 ns, pulse energy 0.1 mJ). A bifurcated fiber bundle (Lot-Oriel LLB321) sent the laser pulse to a single seed put in a dark holder and collected the emitted light. The seed was facing the laser beam in the same configuration used for ion beam irradiation. Spectral components of DL were obtained by using optical broadband (80 nm FWHM) Lot-Oriel filters and detected by a single-photon count photomultiplier (PMT) (Hamamatsu R-7206-1, spectral response 300–850 nm) cooled down to –10°C. An electronic gate enables the PMT to start the photon counting few microseconds after the end of the laser pulse. Previous experiments have shown that the time trend of the DL spectral components from seeds exhibit two different decay slopes in the two regions of the emission spectra at λ < 600 nm and λ > 600 nm respectively. For this reason, only two spectral components characterizing the two regions (460 nm and 645 nm) were measured for all the seeds.

To take into account differences in the size of the illuminated area, due to the not exactly ellipsoidal shape of a seed, DL measurement on each seed was repeated three times, on repositioning the seed from one run to the other.

The last column of Table 1 reports the number of seeds whose DL emission was measured immediately after ion irradiation at different doses.

The results obtained for each dose are reported as average values of the whole set of seeds. Standard errors are also evaluated.

## Results and Discussion

The mung bean seeds irradiated by ion beams exhibited a very high resistance to radiation damages. Indeed, all the seeds irradiated with doses up to 1000 Gy showed a germination capability very close to the control samples ones, while at higher irradiation doses the germination capability dropped to 83%.

**Fig 3:**
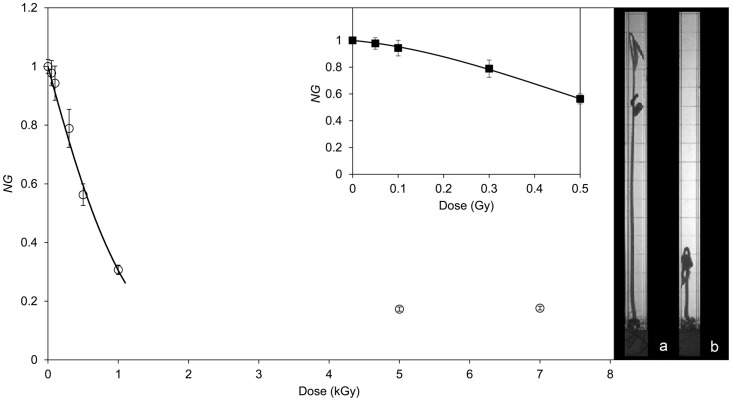
Relation between seedling length and irradiation dose. (○) average values of the normalized seedlings length *NG* as a function of the irradiation dose. (^____^) best fit of the experimental data according to Eq (1). In the little frame the same data at low doses fitted according to Eq (2). Inset: Photograph of a regularly grown (a) and a poorly grown (b) seedling at the same growing day.

Six days after the planting of seeds, the average value of the seedlings length for each dose was evaluated and normalized to the mean length value of control samples. In Fig 3 this parameter (called normalized growth, *NG*) is reported as a function of the ion irradiation dose. The growth was regular for doses up to 300 Gy. Plants were in the vegetative cotyledon stage where unifoliate leaves were unrolled sufficiently so that the leaf edges were not touching, as that shown in the inset (a) in Fig 3. At doses higher than 1000 Gy the germinating seeds either formed only the primary root without any secondary roots or they were in the vegetative emergence stage, characterized by a few roots and a poor evolution of the leaves, where the cotyledons emerged above the hypothetical soil surface and leaflets emerged from cotyledons, as shown in the inset (b) in Fig 3. At intermediate doses both types of growth performance occurred, with a decrease, on increasing the dose, of the number of seeds that showed a regular growth, as that reported in Fig 3 inset (a). More precisely, at 500 Gy irradiation dose only 62% of the seeds grown properly, while such percentage dropped to 24% for seeds irradiated at 1000 Gy (data not shown).

The solid line in Fig 3 represent a fit of the data up to 1000 Gy performed by using the equation:

$$NG=e^{-\alpha \;D}$$ (1)

where *NG* is the normalized growth and *D* the dose measured in Gray. The best fit (reduced χ^2^< 1, R^2^ = 0.990) is obtained when α = (11.4 ± 0.6)×10^−4^. Eq (1) describes the linear dose-response relationship that characterises the effect of densely ionizing radiation on cell survival [10]. Actually in our case, as above said, seeds germination capability remain enough high, while ion irradiation significantly affects seed growth. Interestingly by measuring the effect on the seedlings length the dose response curve Eq (1) is similar to a typical survival curve. Moreover, if one considers only the data up to 500 Gy, we obtained a better fit by using a linear-quadratic model curve

$$NG=e^{-\left(\alpha D\;+\beta \;D^{2}\right)}$$ (2)

with *α* = (3.2 ± 0.6)×10^−4^ and *β* = (1.6 ± 0.1)×10^−6^ (reduced χ^2^< 1, R^2^ = 0.999). The trend of Eq (2) is typical of the response of biological systems to either sparsely ionizing radiations or low linear energy transfer (LET) radiations, which allows that repair mechanism could occur. In our case the ion radiation we used is not considered to have a low LET, nevertheless it crosses freely the seeds and the LET does not change very much (less than 30%) crossing the seed. In addition the low content of water in dry seeds could make such seeds less radiosensitive, so justifying the validity of Eq (2) up to radiation doses of 500 Gy. Consequently, at lower doses, the probability of several damaging events close to each other may be low, as they give mostly easily repairable DNA damage and repair mechanism can occur, while at higher dose, the probability of interacting clustered effects increases, resulting in more severe damage that, in turn, significantly affects the seedlings growth.

**Fig 4:**
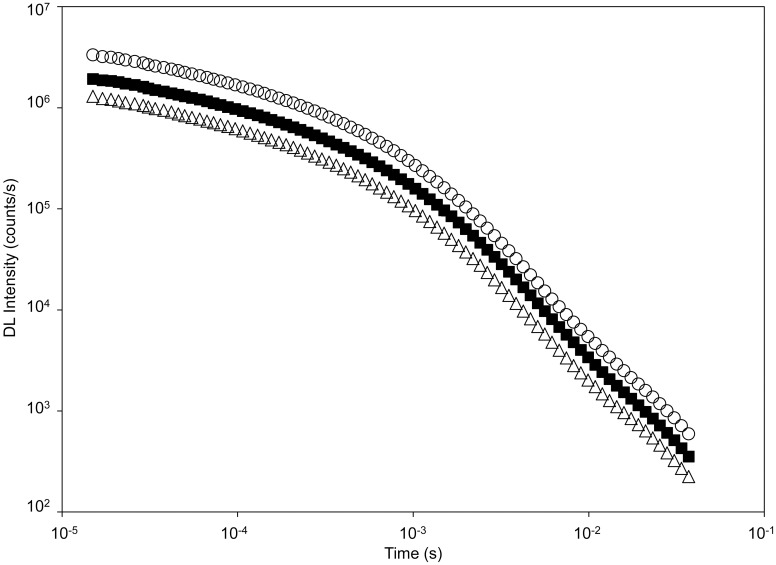
Time trend of Delayed Luminescence emission from seeds. Typical Time trend of the DL emission from a seed at different irradiation doses: (○) native, (■) 100 Gy, (△) 1000 Gy. Data refers to the 450 nm spectral component. Markers denotes average values of 12 seeds. Standard errors are smaller than markers size.

The influence of ion irradiation on DL was also investigated. Fig 4 shows the time trend of the DL spectral component at 450 nm emission wavelength emitted by a single mung bean seed irradiated at 100 Gy and 1000 Gy. For comparison the data of a control seed are also reported.

DL decay trends in Fig 4 show that in the time interval considered hyperbolic curves, which characterize the time relaxation of complex systems [22], better accord the experimental points. Such non-exponentially decay has been correlated to the existence of coherent states [28] which plays an important role in the promotion and control of living systems [29], or to the formation of non-linear coherent self-trapped electron states during charge and energy transfer process in low-dimensional macromolecular structures in cells [26–27].

Differently from what happened in our previous studies, where seeds were damaged with other techniques [23–24], the time trend of DL appears unchanged on increasing the ion irradiation dose. The same happens, on varying the dose, for the spectral component at 650 nm emission wavelength, whose time trend is anyway different from that of the 450 nm one (data not shown). To test that the decay slope does not change with dose we correlated the DL time trend for the irradiated seed to the one of the control sample for each irradiation dose. The best fit of such correlation (data not shown) gave an average slope equal to 1.005±0.007, so confirming the uniformity of the time trends. According to this result, we considered the total number of counts, measured during the time interval ranging from 15 μs up 30 ms, as the DL parameter that is significantly affected by irradiation.

**Fig 5:**
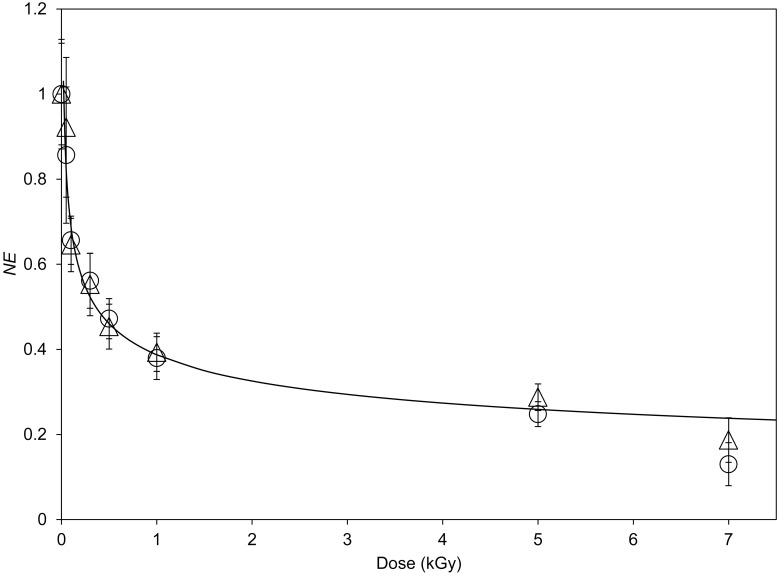
Relation between delayed luminescence yield and irradiation dose. DL total number of counts, normalized to the native ones (*NE*) as a function of the absorbed dose (*D*) for the two spectral components: (○) 450 nm, (△) 650 Gy, (^____^) best fit of the experimental data according to Eq (3). Markers denote average values on the whole seeds data set at every dose (see Table 1). Vertical bars denote standard errors.

Fig 5 shows the total number of emitted photons, normalized to the total number of photons emitted by native seeds (normalized emission, *NE*), as a function of the ion irradiation dose. The data related to the 650 nm spectral emission are reported as triangles, while circles refer to the measurements of the 450 nm spectral emission. For both data sets the correlation can be well accorded to a hyperbolic curve as:

$$NE=b\;D^{-m}$$ (3)

The best fit of the data of both the spectral emissions is obtained when *b* = 2.2 ± 0.3 and *m* = 0.25 ± 0.02 (reduced χ^2^ < 1), when the dose is measured in Gray.

A similar hyperbolic dependence of the total number of counts was measured in the DL of Cadmium sulfide crystals as a function of the crystal volume [25]. In that case we found *m* = 0.33 [25]. For seeds irradiation it is realistic to consider that the impinging ions break the seeds internal structures decreasing their average size, and the relationship between dose and volume is not linear. As a matter of facts, it was observed that the radiation induced in silicon crystals an amorphization of the structure and the ratio between ordered and amorphous volumes depended nonlinearly on dose [30]. The same was observed in the case of radiation damage of protein crystals [31]. So even if one supposes that the DL is connected to the crystal volume by a power law with slope equal to 0.33, by considering the nonlinear dependence of crystal volume on dose, it results reasonable that the correlation between DL and dose is expressed by a power law, having a slope of the order of 0.25. These results suggest that, even in the case of seeds, the ion irradiation produces a decrease in the volume of the organized structures inside the seed and DL from seeds gives information on the evolution of its structural organization.

**Fig 6:**
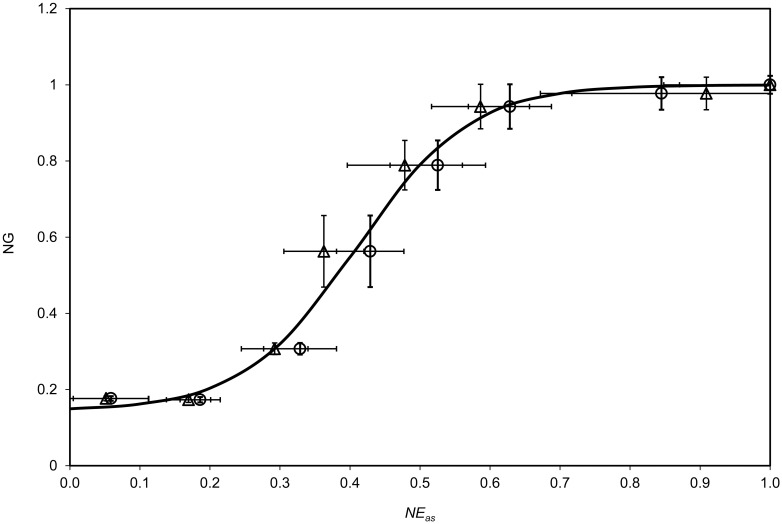
Relation between seedling growth and delayed luminescence yield. Normalized growth NG as a function of the normalized emission NE_as_, after subtraction of DL emission of non-germinating seeds, for the two spectral components at (○) 450 nm, (△) 650 Gy. (^____^) Best fit of both set of data according to Eq (4). Markers denote average values on the whole seeds data set at the same dose. Bars denote standard errors.

As it regards the relation between the seedlings growth and the DL emission, it must be taken into account that also no-germinating seeds show DL emission different from zero. So it is suitable to normalize DL total counts of ion irradiated samples to the DL total counts of native ones after subtraction of DL total counts of no-germinating seeds. In Fig 6 such normalized DL emission (*NE*_*as*_) is reported as a function of the normalized growth *NG*. A single logistic curve:

$$NG=\frac{1+me^{-\;\frac{NE_{as}}{\delta }}}{1+ne^{-\;\frac{NE_{as}}{\delta }}}$$ (4)

is able to accord the data of both 450 nm (dots) and 650 nm (triangles) emission spectral components. The best fit (reduced χ^2^ <1) is reported in Fig 6 as solid line, and it is obtained by:

m = 23 ± 1,

n = 160 ± 6,

$$\delta \;=\;\left(8.07\;\pm \;0.05\right)\;\times \;10^{-2}$$

This logistic equation once again reveals the connection existing between the physiological parameters of the biological systems and the DL and could permit to foreseen the future germination performances of irradiated seeds when they are in the dormant state.

## Conclusions

The effect of irradiation by intermediate energy ion beams on dry mung bean was studied. In particular the seeds were irradiated on air by using a ^12^C^5+^ ion beam at energy of 62 MeV/nucleon, and doses ranged from 50 to 7000 Gy. No less than 24 seeds/dose were analyzed. The development of seedling in first days was measured along with the DL emission from irradiated seeds. Ion irradiation on dry seeds does not significantly affect the germination capability, but slow down the seedlings elongation with blockage of leaves evolution at higher doses.

Correlations among ion dose, seeds growth and DL were studied. Once again the sensitivity of DL to the functional state of single seeds has been demonstrated and a logistic equation was established that could be able to foresee the behavior of seedling starting from DL measurements on dry seed.

This result could be useful in the frame of the development of new techniques to breed plants by using heavy-ion irradiation. Indeed the possibility to know with high probability the evolution of future seedlings could permit to reduce the number of individual seed that should be planted looking for some useful mutation of offspring.

It is interesting to stress that, the analytical dependence of DL from the absorbed dose is correlated to a kind of a structural disorder induced by radiation in native seeds, that is DL gives information on the evolution of the structural organization of the seed after irradiation. This fact, as observed both in condensed and soft matter studies, suggests that DL in biological systems is connected to the existence of collective electronic states whose properties depend on the organized structure of the system. Indeed, such collective excitation of the system, appears to be quenched in damaged seeds.

In conclusion these results suggest that a fast non-invasive single seed quality discrimination technique based on the yield measurements of DL could be used in the field of plants breeding by ion beams. Moreover they suggest that the delayed luminescence could be in general used as a fast and non-invasive technique to check the effects of ion beams on relatively simple biological systems.

## References

[1] Schulz-Ertner D, Tsujii H. Particle radiation therapy using proton and heavier ion beams. J Clin Oncol. 2007; 25(8): 953–64. 10.1200/JCO.2006.09.7816 17350944

[2] Baskar R, Lee KA, Yeo R, Yeoh KW. Cancer and Radiation Therapy: Current Advances and Future Directions. Int J Med Sci. 2012; 9(3):193–9. 10.7150/ijms.3635 22408567 PMC3298009

[3] Liu Y, Liu Y, Sun C, Gan L, Zhang L, Mao A, et al Carbon Ion Radiation Inhibits Glioma and Endothelial Cell Migration Induced by Secreted VEGF. PLoS ONE. 2014; 9(6): e98448 10.1371/journal.pone.0098448 24893038 PMC4043910

[4] Yu Z. Ion beam application in genetic modification. IEEE T Plasma Sci. 2000; 28(1): 128–32.

[5] Abe T, Matsuyama T, Sekido S, Yamaguchi I, Yoshida S, Kameya T. Chlorophyll-deficient Mutants of Rice Demonstrated the Deletion of a DNA Fragment by Heavy-ion Irradiation. J Radiat Res.2002; 43: S157–S161. 12793751 10.1269/jrr.43.s157

[6] Morishita T, Yamaguchi H, Degi K, Shikazono N, Hase Y, Tanaka A, et al Dose response and mutation induction by ion beam irradiation in buckwheat. Nucl Instrum Meth B. 2003; 206: 565–9.

[7] Honda I, Kikuchi K, Matsuo S, Fukuda M, Saito H, Ryuto H, et al Heavy-ion-induced mutants in sweet pepper isolated by M1 plant selection. Euphytica. 2006; 152(1): 61–6.

[8] Lodge M, Pijls-Johannesma M, Stirk L, Munro AJ, De Ruysscher D, Jefferson T. A systematic literature review of the clinical and cost-effectiveness of hadron therapy in cancer. Radiother Oncol. 2007; 83(2): 110–122. 10.1016/j.radonc.2007.04.007 17502116

[9] Choi J, Kang JO. Basics of particle therapy II: relative biological effectiveness. Radiat Oncol J. 2012; 30(1):1–13. 10.3857/roj.2012.30.1.1 23120738 PMC3475957

[10] Habermehl D, Ilicic K, Dehne S, Rieken S, Orschiedt L, Brons S, et al The Relative Biological Effectiveness for Carbon and Oxygen Ion Beams Using the Raster-Scanning Technique in Hepatocellular Carcinoma Cell Lines. PLoS ONE. 2014; 9(12): e113591 10.1371/journal.pone.0113591 25460352 PMC4252049

[11] Blakely EA, Chang PY. Late effects from hadron therapy. Radiother Oncol. 2004; 73: S134–S140. 15971329 10.1016/s0167-8140(04)80035-5

[12] Zhang B, Liu B, Zhang H, Wang J. Erythrocyte Stiffness during Morphological Remodeling Induced by Carbon Ion Radiation. PLoS ONE. 2014; 9(11): e112624 10.1371/journal.pone.0112624 25401336 PMC4234377

[13] Wang TJC, Wu C-C, Chai Y, Lam RKK, Hamada N, Kakinuma S, et al Induction of Non-Targeted Stress Responses in Mammary Tissues by Heavy Ions. PLoS ONE. 2015; 10(8): e0136307 10.1371/journal.pone.0136307 26317641 PMC4552651

[14] Hsu BD. On the possibility of using a chlorophyll fluorescence parameter as an indirect indicator for the growth of Phalaenopsis seedlings. Plant Sci. 2007; 172: 604–8.

[15] Subedi PP, Walsh KB, Owens G. Prediction of mango eating quality at harvest using short-wave near infrared spectrometry. Postharvest Biol Techn. 2007; 43: 326–34.

[16] Zude M, Birlouez-Aragon I, Paschold PJ, Rutledge DN. Non-invasive spectrophotometric sensing of carrot quality from harvest to consumption. Postharvest Biol Techn. 2007; 45: 30–7.

[17] Saeys W, Velazco-Roa MA, Thennadil SN, Ramon H, Nicolaï BM. Optical properties of apple skin and flesh in the wavelength range from 350 to 2200 nm. Appl Opt. 2008; 47(7): 908–19. 18311262 10.1364/ao.47.000908

[18] Niggli HJ. Ultraweak Electromagnetic Wavelength Radiation as Biophotonic Signals to Regulate Life. J Electr Electron Syst. 2014; 3(2): 126.

[19] Niggli HJ, Tudisco S, Privitera G, Applegate LA, Scordino A, Musumeci F. Laser-ultraviolet-A-induced ultraweak photon emission in mammalian cells. J Biomed Opt. 2005; 10: 024006 10.1117/1.1899185 15910080

[20] Scordino A, Campisi A, Grasso R, Bonfanti R, Gulino M, Iauk L, et al Delayed luminescence to monitor programmed cell death induced by berberine on thyroid cancer cells. J Biomed Opt. 2014; 19 (11): 117005 10.1117/1.JBO.19.11.117005 25393968

[21] Baran I, Ionescu D, Privitera S, Scordino A, Mocanu MM, Musumeci F, et al Mitochondrial respiratory complex I probed by delayed luminescence spectroscopy. J BIOMED OPT. 2013; 18(12): 127006 10.1117/1.JBO.18.12.127006 24365956

[22] Scordino A, Baran I, Gulino M, Ganea C, Grasso R, Niggli HJ, et al Ultra-weak delayed luminescence in cancer research: A review of the results by the ARETUSA equipment. J Photoch Photobio B. 2014; 139: 76–84. 10.1016/j.jphotobiol.2014.03.027 24912405

[23] Costanzo E, Gulino M, Lanzanò L, Musumeci F, Scordino A, Tudisco S, et al Single seed viability checked by delayed luminescence. Eur Biophys J. 2008; 37: 235–8. 10.1007/s00249-007-0221-8 17952430

[24] Lanzanò L, Sui L, Costanzo E, Gulino M, Scordino A, Tudisco S, et al Time-resolved spectral measurements of delayed luminescence from a single soybean seed: effects of thermal damage and correlation with germination performance. Luminescence. 2009; 24: 409–15. 10.1002/bio.1127 19424957

[25] Scordino A, Triglia A, Musumeci F. Analogous features of delayed luminescence from Acetabularia acetabulum and some solid state systems. J Photoch Photobio B. 2000; 56: 181–6. 10.1016/s1011-1344(00)00078-6 11079479

[26] Brizhik L, Scordino A, Triglia A, Musumeci F. Delayed luminescence of biological systems arising from correlated many-soliton states. Phys Rev E. 2001; 64(3): 031902. 10.1103/PhysRevE.64.031902 11580362

[27] Brizhik L, Musumeci F, Scordino A, Tedesco M, Triglia A. Nonlinear dependence of the delayed luminescence yield on the intensity of irradiation in the framework of a correlated soliton model. Physical Review E. 2003; 67: 021902. 10.1103/PhysRevE.67.021902 12636710

[28] Popp FA, Li KH. Non-exponential decay law of radiation systems with coherent rescattering. Phys Lett A. 1983; 93(5): 262–266.

[29] Ho MW. Bioenergetics and the coherence of organisms. Neuronetwork World. 1995; 5: 733–750.

[30] Pelaz L, Marqués LA, Barbolla J. Ion-beam-induced amorphization and recrystallization in silicon. J Appl Phys. 2004; 96: 5947.

[31] Southworth-Davies R J, Medina MA, Carmichael I, Garman EF. Observation of decreased radiation damage at higher dose rates in room temperature protein crystallography. Structure. 2007; 15 (12): 1531–41. 10.1016/j.str.2007.10.013 18073104

